# Clinicopathological Features and Prognostic Factors Affecting Survival Outcomes in Isolated Locoregional Recurrence of Breast Cancer: Single-Institutional Series

**DOI:** 10.1371/journal.pone.0163254

**Published:** 2016-09-20

**Authors:** Min-Young Lee, Won Jin Chang, Hae Su Kim, Ji Yun Lee, Sung Hee Lim, Jeong Eon Lee, Seok Won Kim, Seok Jin Nam, Jin Seok Ahn, Young-Hyuck Im, Yeon Hee Park

**Affiliations:** 1 Division of Hematology and Oncology, Department of Medicine, Samsung Medical Center, Sungkyunkwan University School of Medicine, Seoul, Korea; 2 Department of Surgery, Samsung Medical Center, Sungkyunkwan University School of Medicine, Seoul, Korea; Virginia Mason Medical Center, UNITED STATES

## Abstract

**Purpose:**

The purpose of this study was to investigate the clinicopathologic features and prognostic factors affecting outcome in patients with isolated locoregional recurrence of breast cancer (ILRR).

**Methods:**

We retrospectively analyzed the medical records of 104 patients who were diagnosed with ILRR and underwent curative surgery from January 2000 to December 2010 at Samsung Medical Center.

**Results:**

Among 104 patients, 43 (41%) underwent total mastectomy and 61 (59%) underwent breast-conserving surgery for primary breast cancer. The median time from initial operation to ILRR was 35.7 months (4.5–132.3 months). After diagnosis of ILRR, 45 (43%) patients were treated with mastectomy, 41 (39%) with excision of recurred lesion, and 18 (17%) with node dissection. During a median follow-up of 8.9 years, the 5-year overall survival was 77% and 5-year distant metastasis-free survival (DMFS) was 54%. On multivariate analysis, younger age (< 35 years), higher stage, early onset of elapse (≤ 24 months), lymph node recurrences, and subtype of triple negative breast cancer (TNBC) were found to be independently associated with DMFS. Patients in the no chemotherapy group showed a longer DMFS after surgery for ILRR than those treated with chemotherapy (median 101.5 vs. 48.0 months, p = 0.072) but without statistical significance.

**Conclusion:**

Our analysis showed that younger age (< 35 years), higher stage, early onset of relapse (≤ 24 months), lymph node recurrence, and subtype of TNBC are the worst prognostic factors for ILRR.

## Introduction

Isolated locoregional recurrence (ILRR) of breast cancer after breast-conserving surgery (BCS) or mastectomy is associated with an increased risk of distant metastases and a poor prognosis [1–6]. The incidence of ILRR is approximately 10–13% after BCS and 3–8% after mastectomy [7,8]. For patients with recurrence, salvage mastectomy has been the predominant local treatment modality for most patients with operable ILRR but second BCS might be considered in some patients, particularly those with small and late recurrence [9–11]. Furthermore, these patients are regarded candidates for subsequent adjuvant systemic treatments with curative intent. However, the role of systemic adjuvant treatment in the management of ILRR is not well established [12,13]. In addition, although there are data on salvage surgery, systemic chemotherapy, and hormonal therapy, treatment guidelines for ILRR and the best strategy for patients with ILRR remain controversial. This is because patients have heterogeneous biological features and receive different initial therapies according to disease status of node positive or negative and hormonal receptor positive or negative, therefore analysis of the clinical data of various patients with ILRR is required. The aim of this study was to analyze clinicopathologic features and investigate prognostic factors of outcome in a single-institution series of patients with ILRR.

## Materials and Methods

### Patients

From January 2000 to December 2010, 4,700 patients received curative surgery for breast cancer at Samsung Medical Center. We conducted a retrospective analysis of the medical records of patients with operable isolated locoregional recurrence (ILRR) with negative resection margin as a first event and no evidence of synchronous metastatic disease. ILRR after mastectomy was defined as initial reappearance of cancer in either the ipsilateral skin or chest wall, or cervical, internal mammary, supraclavicular, infraclavicular, or axillary nodes on ipsilateral side. ILRR after BCS included definition about the appearance of tumor in the same quadrant as the initial tumor. Patients diagnosed after distant metastases or with synchronous distant disease were excluded from this analysis.

### Data collection

We collected demographic data and treatment details as follows: age, gender, date of surgery, method of surgery, presenting features of the ILRR, treatment modalities used (chemotherapy, radiotherapy, or hormonal therapy), and clinical course such as time to ILRR, distant progression, and survival. Clinicopathological data including histology, pathologic stage (American Joint Committee on Cancer 6^th^ edition, AJCC), nuclear grade, tumor size, margin status, and status of the estrogen receptor (ER), progesterone receptor (PgR), and human epidermal growth factor receptor type 2 (HER2), as assessed using immunohistochemical (IHC) staining, were reviewed. The nodal status was also classified according to AJCC. The pN+ (pN1, pN2, and pN3) included micrometastases in lymph nodes (pN1mi, defined as greater than 0.2 mm and/or more than 200 cells, but none greater than 2.0 mm) and the pN- (pN0) included isolated tumor cells (ITC, defined as small clusters of cells not greater than 0.2 mm, or single tumor cells, or a cluster of fewer than 200 cells in a single histologic cross-section) in completion axillary dissection. ER and PgR positivity was defined as an Allred score of 3–8 by IHC using antibodies to ER (Immunotech, Marseille, France) and PgR (Novocastra, Newcastle upon Tyne, UK). HER2 status was evaluated using an anti-HER2 antibody (DAKO, Glostrup, Denmark) and/or fluorescence in situ hybridization (FISH). This study was approved by the Institutional Review Board of Samsung Medical Center, Seoul, South Korea. Informed consent was waived because the study was based on retrospective clinical data.

### Statistical analysis

Distant metastasis-free survival (DMFS) was defined as the time from the first ILRR to the first appearance of distant metastasis. Overall survival (OS) was defined as survival from the date of first ILRR to death from or the last follow-up. The Kaplan-Meier methodology was used to estimate survival probability, which was expressed as a mean with a range and two-sided 95% confidence interval (CI) and compared between two or more groups of patients using the log-rank test. Cox proportional hazards regression models were used to identify independent factors associated with DMFS or OS. Comparisons of categorical variables among groups were evaluated using either the Pearson’s Chi-square test or Fisher exact test. A two-sided p-value less than 0.05 was considered statistically significant. All statistical analyses were performed using a software package (SPSS, Version 18.0 Network version, Inc., Chicago, IL, USA).

## Results

### Patient cohort

**Fig 1:**
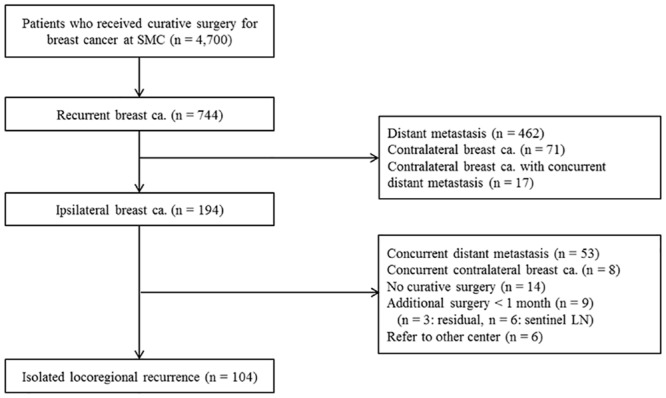
Patient cohort. SMC, Samsung Medical Center; LN, lymph node.

We retrospectively reviewed medical records for 4,700 patients with histologically confirmed breast cancer who underwent curative surgery at Samsung Medical Center from January 2000 to December 2010. Of these 4,700 patients, 744 (15.8%) had recurrent breast cancer and 194 (4.1%) had an ipsilateral breast recurrence as a first event during regular follow-up. We excluded 53 patients who had concurrent distant metastasis, 8 with concurrent contralateral breast cancer, 14 who did not receive curative surgery, 9 who had an additional surgery within at least 1 month, and 6 who were referred to other centers (Fig 1). ILRR occurred in 104 patients, for an overall ILRR rate of 2.2%.

### Clinicopathological characteristics of patients with ILRR

Patient characteristics are summarized in Table 1 (at presentation of primary breast cancer) and Table 2 (at the time of ILRR). The median age at initial diagnosis was 46 years (range, 27–76 years). Most patients (82.7%) had stage I or stage II disease. Of these 104 patients, 56 (53.8%) were HR+/HER2- (defined as ER+ and/or PgR+, and HER2-), 26 (25.0%) were HER2+ (defined as HER2+, irrespective of ER or PgR status), and 22 (21.2%) were triple-negative breast cancers (TNBC, defined as ER-, PgR-, and HER2-) at the time of primary breast cancer diagnosis. All patients underwent curative surgery: 43 (41.3%) underwent total mastectomy and 61 (58.7%) patients underwent breast-conserving surgery for the initial presenting breast cancer. The postoperative pathology revealed ductal carcinoma in 92 of 104 patients (88.5%) and non-ductal carcinoma in 12 (11.5%) patients (papillary carcinoma in 5, mucinous carcinoma in 2, mixed carcinoma in 2, medullary carcinoma in 1, cribriform carcinoma in 1, tubular carcinoma in 1 patient). Lymph nodes were pathologically positive in 41 patients (40.4%). Adjuvant treatment consisted of chemotherapy in 76.9% of the patients, adjuvant radiotherapy in 73.1%, and hormonal therapy in 59.6%. Systemic chemotherapy regimens for primary breast cancer were as follows: 26 patients received anthracycline-based chemotherapy, 32 patients received anthracycline-based chemotherapy followed by taxane, and 22 patients received cyclophosphamide/methotrexate/fluorouracil combination chemotherapy. Only five patients received HER2 combination chemotherapy for primary breast cancer because HER2-directed therapy was not available until 2010 in our institute. Among the patients with HR+ (defined as ER+ and/or PgR+), most of the patients (60 of 65 patients, 92.3%) received adjuvant hormonal therapy as follows: tamoxifen in 47, non-steroidal aromatase inhibitor in 12, steroidal aromatase inhibitor in 1 patient.

**Table 1:** Characteristics of patients at the time of primary diagnosis (n = 104).

Characteristics	Number	%
Age (median, range)	46 (27–76)	
< 40	29	27.9
40–50	46	44.2
> 50	29	27.9
Primary surgery type		
Mastectomy	43	41.3
Breast conserving surgery	61	58.7
Histology		
Ductal	92	88.5
Non-ductal	12	11.5
Tumor nuclear grade		
Low	10	9.6
Intermediate	41	39.4
High	49	47.1
Undescribed	4	3.8
Tumor size (pT)		
pT1 (Tumor ≤ 20mm)	62	59.6
pT2-pT3 (Tumor > 20mm)	41	39.4
Nodal status (pN)		
pN- (pN0)	62	59.6
pN+ (pN1, pN2, pN3)	41	40.4
Stage		
IA	45	43.3
IIA-IIB	41	39.4
IIIA-IIIC	18	17.3
Subtype		
HR+/HER2-	56	53.8
HER2+, irrespective of HR+/-	26	25.0
TNBC	22	21.2
Treatment of primary tumor		
Adjuvant chemotherapy	80	76.9
Adjuvant radiotherapy	76	73.1
Adjuvant hormonal therapy	62	59.6

HR, hormone receptor; HER2, human epidermal growth factor receptor 2; TNBC, Triple negative breast cancer

**Table 2:** Characteristics of patients at the time of ILRR (n = 104).

Characteristics	Number	%
Time from primary surgery to ILRR		
Median, range (month)	35.7 (4.5–132.3)	
Time from ILRR to distant metastasis	n = 49	
Median, range (month)	17.9 (1.5–111.1)	
Location of ILRR		
Breast	56	53.8
Surgery scar or chest wall	13	12.5
Regional lymph node	35	33.7
Histology		
Ductal	88	84.6
Non-ductal	16	15.4
Subtype		
HR+/HER2-	35	33.7
HER2+, irrespective of HR+/-	36	34.6
TNBC	15	14.4
Not checked	18	17.3
Surgical treatment		
Mastectomy	45	43.3
Excision	41	39.4
Node dissection	18	17.3
Treatment of ILRR		
Chemotherapy	31	29.8
Radiotherapy	36	34.6
Hormonal therapy	47	45.2

ILRR, isolated locoregional recurrence; HR, hormone receptor; HER2, human epidermal growth factor receptor 2; TNBC, Triple negative breast cancer

Of the total study population, the median time from initial curative surgery to ILRR was 35.7 months (range, 4.5–132.3 months). The median age at occurrence of ILRR was 49 years (range, 27–60 years). The site of locoregional recurrence was at the breast in 56 patients (53.8%), at the chest wall or incision scar in 13 patients (12.5%), and at the regional lymph nodes in 35 patients (33.7%). All patients also underwent curative surgery for ILRR: 45 patients (43.3%) were treated with mastectomy, 41 (39.4%) with excision of recurred lesion, and 18 (17.3%) with node dissection. Among the patients recurred in breast, 38 patients performed mastectomy and 18 patients treated with wide excision of recurred breast lesion. In these patients, 8 patients (44.4%) attempt second BCS at the time of ILRR despite previously performed BCS. HR and HER2 status was evaluated in 86 patients. For 18 patients who showed a change in HR status (ER or PgR), the HR status changed from positive to negative in 10 patients and from negative to positive in 8 patients. Among the 21 patients with changes in HER2 expression, 5 changed from HER2 positive to negative and 16 from negative to positive (Table 3). Therefore, of the total 104 patients, 35 (33.7%) patients were HR+/HER2-, 36 (34.6%) were HER2+, and 15 (14.4%) were TNBC at the time of ILRR diagnosis. After the operation for ILRR, systemic chemotherapy was administered to 29.8% of the patients, radiotherapy to 34.6%, and hormonal therapy to 45.2%. Systemic chemotherapy regimens for ILRR were selected by the treating physicians for each individual patient on the basis of disease characteristics and previous therapies for the primary breast cancer. Most of the patients (25 of 31 patients, 80.6%) received combination chemotherapy as follows: anthracycline-based in 12, taxane-based in 11, anthracycline plus taxane-based combination chemotherapy in 2 patients. Of the 6 patients treated with monochemotherapy, 4 received capecitabine and 2 received taxane. Among these patients, 10 also received HER2 therapy.

**Table 3:** Changes of HR status and HER2 expression on IHC stain.

Biologic marker	Number	%
HR status		
Change	18	
(+) → (-)	10	15.4
(-) → (+)	8	20.5
HER2 expression		
Change	21	
(+) → (-)	5	19.2
(-) → (+)	16	20.5
TNBC → TNBC	9	40.9
TNBC → HR+ or HER2+	10	45.5
HR+ or HER2+ → TNBC	6	7.3

IHC, immunohistochemical; HR, hormone receptor; HER2, human epidermal growth factor receptor 2; TNBC, Triple negative breast cancer

### Distant metastasis rate and survival outcomes for ILRR

The median follow-up duration from date of diagnosis of ILRR was 8.9 years (range, 1.1–14.5 years). Twenty-eight of the 104 patients (26.9%) died during follow-up, with OS at 5 years of 77%. We observed an increase in OS with increasing interval time to ILRR (≤ 24 vs. 25–48 vs. > 48 months; log-rank p value 0.001). Overall, 49 patients (47.1%) had relapsed disease during the follow-up, with a distant metastasis-free survival (DMFS) rate at 5 years of 54%. The median DMFS was 5.5 years (95% CI: 1.5–9.4). Again, we observed an increasing DMFS with increasing interval time to ILRR (≤ 24 vs. 25–48 vs. > 48 months; log-rank p value 0.010).

**Fig 2:**
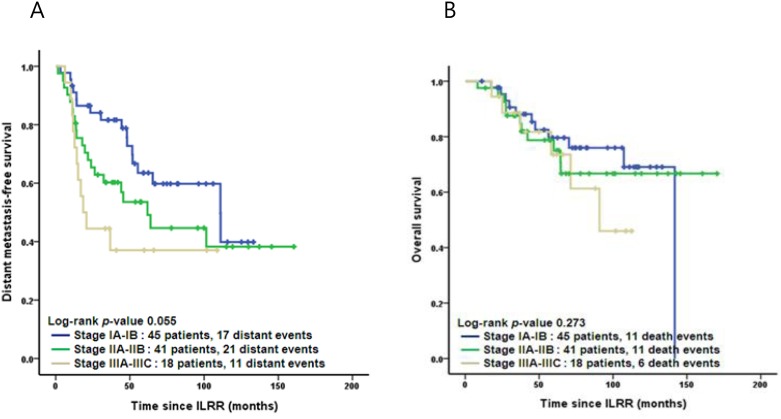
Kaplan-Meier curves: Distant metastasis-free (A) and overall (B) survival by the stage. ILRR, isolated locoregional recurrence.

**Fig 3:**
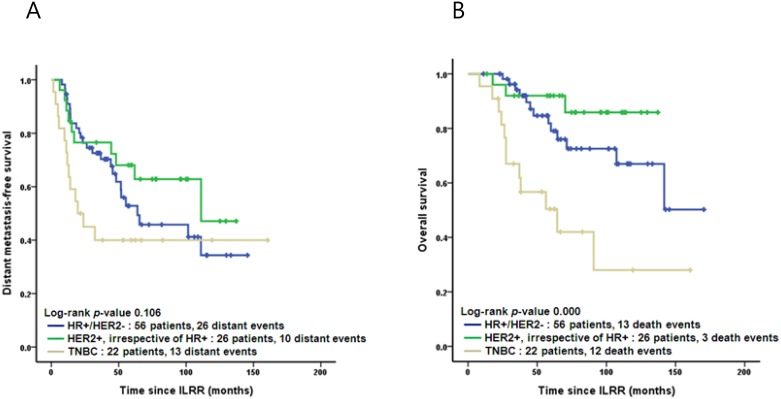
Kaplan-Meier curves: Distant metastasis-free (A) and overall (B) survival by tumor subtype. ILRR, isolated locoregional recurrence; HR, hormone receptor; HER2, human epidermal growth factor receptor 2; TNBC, Triple negative breast cancer.

The Kaplan-Meier curves for DMFS and OS by stage and subtype are shown in Figs 2 and 3. Tables 4 and 5 list the results of multivariate analysis to examine the influence of a variety of factors on DMFS and OS. In the Cox regression analysis, patients with TNBC subtype had the worst DMFS (hazard ratio [HR], 3.183; 95% CI, 1.503–6.739; p = 0.002) and worst OS (HR, 4.057; 95% CI, 1.707–9.642; p = 0.002), when compared with subtype HR+/HER2- and HER2+. Adjustment was made for age, previous surgery methods, stage, time interval to ILRR, recurrence location of ILRR, and adjuvant chemotherapy for ILRR.

**Table 4:** Cox regression multivariate analysis for distant metastasis.

Variable	Significance (p-value)	Hazard ratio	95% CI for Exp (B)	
			Lower	Upper
Age ≥ 35 years	0.004	0.371	0.188	0.731
Mastectomy (vs BCS)	0.532	1.249	0.622	2.507
Stage III (vs Stage I & II)	0.040	2.220	1.039	4.746
TNBC (vs HR+ & HER2+)	0.002	3.183	1.503	6.739
Interval > 24 months	0.006	0.410	0.218	0.772
Recurrence site at LNs (vs breast & chest wall)	0.006	2.816	1.342	5.908
Adjuvant chemotherapy (vs no chemotherapy)	0.391	1.329	0.694	2.545

CI, confidential interval; BCS, breast conserving surgery; HR, hormone receptor; HER2, human epidermal growth factor receptor 2; TNBC, Triple negative breast cancer; LN, lymphnode

**Table 5:** Cox regression multivariate analysis for overall survival.

Variable	Significance (p-value)	Hazard ratio	95% CI for Exp (B)	
			Lower	Upper
Age ≥ 35 years	0.370	0.625	0.223	1.748
Mastectomy (vs BCS)	0.256	0.554	0.200	1.534
Stage III (vs Stage I & II)	0.078	2.623	0.897	7.669
TNBC (vs HR+ & HER2+)	0.002	4.057	1.707	9.642
Interval > 24 months	0.002	0.252	0.105	0.603
Recurrence site at LNs (vs breast & chest wall)	0.052	2.518	0.993	6.382
Adjuvant chemotherapy (vs no chemotherapy)	0.035	2.367	1.064	5.268

CI, confidential interval; BCS, breast conserving surgery; HR, hormone receptor; HER2, human epidermal growth factor receptor 2; TNBC, Triple negative breast cancer; LN, lymphnode

DMFS after the operation for ILRR was longer in the no chemotherapy group than in the group that received adjuvant chemotherapy (median 101.5 vs. 48.0 months, p = 0.072), but without statistical significance. In analysis according to treatment group, there was significant difference about surgical procedure performed for primary and recurred tumors in patients treated systemic chemotherapy or radiotherapy after the diagnosis of ILRR. In chemotherapy group, patients received BCS more than mastectomy (p = 0.036) for primary breast cancer, and mastectomy more than excision or node dissection (p = 0.004) for ILRR. In radiotherapy group, patients underwent mastectomy more than BCS (p = 0.000) for primary breast cancer, and excision or node dissection more than mastectomy (p = 0.000) for ILRR. Other clinicopathological characteristics were not significant differences in each group.

## Discussion

The incidence of ILRR of breast cancer is approximately 10–13% within 10 years after BCS, and 3–8% after mastectomy plus postoperative radiotherapy [7,8]. Despite local treatment, ILRR has been associated with an increased risk of distant metastasis and poor prognosis [2,3,6,14,15]. The disease-free interval is one of the most powerful prognostic factors of survival and the critical time interval to recurrence is associated with the highest risk of subsequent distant metastasis and death [2,6,16,17]. In the EORTC 10801 trial, van Dongen et al. [18] observed that 73% of patients with ILRR < 2 years after mastectomy had subsequent distant metastasis, compared to 35% with an interval ≥ 2 years.

There are still no standard guidelines for treatment strategies after ILRR. Several studies have reported salvage surgery as a therapeutic modality available to patients with ILRR. The generally recommended treatment for ILRR after BCS is salvage mastectomy, although the outcome of a second conservative procedure has also recently been investigated. In a retrospective analysis from the European Institute of Oncology [9], 161 patients with ILRR underwent a second BCS with 5-year cumulative incidence of further recurrence of the tumor after second BCS of 15.2% in the subset of patients with a recurrent tumor < 2 cm that occurred more than 48 months after the primary cancer treatment. There are few studies of the role of salvage hormonal therapy. Results of the randomized phase III SAKK 23/82 trial comparing tamoxifen to observation demonstrated that tamoxifen significantly improved the post-recurrence DFS for patients with ER-positive tumors. In long-term analysis at a median follow-up of 11.6 years, this trial showed a 5-year DFS of 40% in the observation group and 61% in the tamoxifen group. Another salvage treatment option is systemic chemotherapy. The CALOR trial was the first randomized trial to investigate the use of chemotherapy in patients with ILRR [19]. In this trial, 162 patients with completely excised ILRR were randomly allocated to either chemotherapy or no chemotherapy. The 5-year disease free-survival (DFS) was 69% in the chemotherapy group compared with 57% in the no chemotherapy group (p = 0.046). Adjuvant chemotherapy also resulted in an improved 5-year survival of 88% versus 76% (p = 0.024). In particular, this study also showed that chemotherapy was significantly more effective for patients in the ER-negative group. Although these studies support a potential role for adjuvant hormonal therapy and chemotherapy in ILRR, the precise procedure and optimal treatment remain uncertain.

In our study, patients with operable ILRR had 5-year OS of 77% and 5-year DMFS of 54% during the median follow-up duration of 8.9 years. ILRR that occurred later during follow-up was associated with better prognosis than that occurring earlier (≤ 24 vs. 25–48 vs. and > 48 months). In addition, among women diagnosed with ILRR, younger age (< 35 years), higher stage, early onset of the relapse (≤ 24 months), lymph node recurrences, and subtype of TNBC were independent factors affecting distant metastasis in our cohort. Likewise, early onset of the relapse (≤ 24 months), and subtype of TNBC were independent factors for survival rate. Survival analysis using the Cox proportional hazards method suggested that tumor subtype had a higher hazard ratio (DMFS, HR 3.183, 95% CI, 1.503–6.739; OS, HR 4.057; 95% CI, 1.707–9.642) than other variables, especially stage. The patients with TNBC showed significant lower DMFS and OS (median DMFS, 101.5 vs 19.6 months; median OS, not reached vs 64.4 months) compared with the other groups. This implies that the influence of tumor subtype on overall survival rate is so large that the effect of disease can essentially be ignored. In these factors affecting DMFS and OS, it is also well known that women with TNBC has been associated with younger age, more advanced stage and have early relapse in epidemiological research [20]. However, the patients with TNBC tend to have early recurrence, but they did not show a tendency to younger age, higher stage or node recurrence, in our study populations. This difference is due to the retrospective analysis that TNBC group is a small number of included in this study than in the other group. Several prospective and large-cohort retrospective studies have demonstrated that alterations in HR and HER2 status between individual primary tumors and relapsed tumors have statistically significant prognostic implications [21,22,23]. Our data showed similar results, indicating the importance of salvage treatment according to HR and HER2 status of ILRR.

The present study had several limitations. First, our study was a retrospective analysis performed at a single institution and there might be a selection bias of physicians concerning which individual patients underwent salvage treatment. There is also a limitation to interpreting our results because a variety of salvage chemotherapy regimens and hormonal therapy drugs were used. In addition, the patients who have not received HER2-directed therapy included our study cohort even though they have HER2-overexpressing tumor because adjuvant HER2-directed therapy has been reimbursed since 2010 in Korea. At presentation of primary breast cancer, only small proportion of the patients (5 of 18, 27.7%) could have received HER2-directed therapy among the patients who underwent systemic chemotherapy in patients with HER2 positive. However, most of the patients (10 of 12, 83.3%) have received HER2 therapy among the patients who underwent systemic chemotherapy in HER2+ expression at the time of ILRR. This limitation may have affected our findings. Second, the ILRR patient group had a small sample size compared to the incidence of ILRR in other reported studies. This low incidence and small number of patients who were diagnosed with ILRR limited our ability to identify meaningful predictive or prognostic factors. Third, our study did not clearly classified ILRR as either true local recurrence or new primary tumors. Several studies have attempted to classify these two entities by the location, histology, DNA flow cytometry, and/or molecular criteria [24,25]. This distinction is important because it could be related different natural histories, prognosis, or different implications for therapeutic management. However, several studies used various criteria because of the exact criteria to distinct these two entities is not well defined. In this study, we classified ILRR mainly based on tumor location without molecular or genetic sequencing confirmation. In addition, our study populations had a change in histology type in 8 patients (7.7%). Previous reports supported that new primary tumors had better survival rates than true local recurrence. This limitation might be affected to interpreting our results of survival rates.

These limitations might explain why our results for the role of chemotherapy are different from those of previous studies. Among the 104 patients enrolled in our study, DMFS after the operation for ILRR was longer in the no chemotherapy group than in the adjuvant chemotherapy group. This might be because our study was a retrospective review and analyzed patients with ILRR regardless of previous treatment for the primary breast cancer. In the subgroup analysis, there was a significant difference in the type of surgical treatment performed for both the primary tumor and ILRR between patients treated with salvage chemotherapy versus radiotherapy. In addition, we analyzed the role of chemotherapy for each subtype. Among the 22 patients with TNBC, 19 received adjuvant chemotherapy and 3 received no chemotherapy at the time of primary breast cancer diagnosis. No significant differences in distant metastasis-free and overall survival benefits were observed in the chemotherapy groups (log-rank p value 0.364 and 0.254), but this may be due to the small number of patients in each subgroup. We also analyzed the role of each surgical treatment for ILRR. About 43.3% patients performed mastectomy and 56.7% patients treated with excision of recurred lesion or lymph node dissection at presentation of ILRR. Second BCS performed 8 patients (44.4%) in the patients group with previously performed BCS for primary breast cancer. In survival analysis, DMFS rate of the excision or node dissection group was significant lower (p = 0.039) when compared to mastectomy group, but not in OS.

The findings of the current study suggest that it is difficult to evaluate retrospective analyses of ILRR even if a large uniform sample size is obtained. The incidence of ILRR was definitely not low, but the patient population included various patient groups according to biological characteristics of primary breast cancer and heterogeneity of treatment received. In several studies of ILRR, even the recent prospective CALOR study [19], the patients did not receive a unified salvage therapy because the physicians selected personalized surgery, chemotherapy, radiotherapy, or hormonal therapy regimens for their patients on the basis of previously received treatment and changes in biological marker status. Also, recently published research results about HER2 status and HER2-directed therapy such as trastuzumab have become available since 2004 or 2005 and a variety of HER2 directed therapies with trastuzumab, pertuzumab, and T-DM1 are now available. Thus, further studies of ILRR should consider these newly available agents.

In summary, our retrospective review highlights the difficulties associated with analysis of ILRR. ILRR of breast cancer exhibits very heterogeneous characteristics such as alterations in biological markers and variations in treatment modalities involving multiple chemotherapy regimens or hormonal therapy drugs, including newly developed or approved agents. The management of each patient requires a multidisciplinary approach that depends not only on factors specific to the recurrence itself but also on factors related to the original treatment. Future validation in a large prospective series and additional research on proper therapeutic strategies for ILRR with new agents is warranted.

## Conclusions

Our analysis showed that younger age (< 35 years), higher stage, early onset of relapse (≤ 24 months), lymph node recurrence, and subtype of TNBC are the worst prognostic factors for patients with ILRR.
